# Annotated catalogue of the Haliplidae of China with the description of a new species and new records from China (Coleoptera, Adephaga)

**DOI:** 10.3897/zookeys.133.1642

**Published:** 2011-10-05

**Authors:** Fenglong Jia, Bernhard van Vondel

**Affiliations:** 1 Institute of Entomology, Life Science School, Sun Yat-sen University, Guangdong, China; 2 Natural History Museum Rotterdam. p/o: Roestuin 78, 3343 CV Hendrik-Ido-Ambacht, the Netherlands

**Keywords:** Coleoptera, Haliplidae, *Haliplus*, new species, new records, China

## Abstract

A revised checklist of Haliplidae (Coleoptera: Adephaga) of China is presented. A new species *Haliplus (Haliplus) latreillei* **sp. n.** is described from Guizhou, China. Three species, *Haliplus (Haliplidius) confinis* Stephens, *Haliplus (Haliplus) ruficollis* (De Geer) and *Haliplus (Haliplus) sibricus* Motschulsky are reported from China for the first time. *Haliplus dalmatinus* Müller is excluded from the list of Chinese species. A number of new provincial records from China is presented.

## Introduction

Haliplidae is a very small family of Coleoptera. A total of five genera and 238 species (Vondel 2005, 2007, 2009, 2010, Vondel and Spangler 2008, Watts and McRae 2010) have been described in the world of which only *Haliplus* Latreille, 1802 (with subgenera *Liaphlus* Guignot, 1928, *Haliplidius* Guignot, 1928 and *Haliplus* s. str.) and *Peltodytes* Régimbart, 1879 (subgenus *Peltodytes* s.str.) are known in China. Wu (1937) reported two genera and 13 species from China, of which only 10 species are valid now. Since the description of *Haliplus diruptus* Balfour-Browne, 1947 from Tianjin, no new species were described from China until Vondel (1990) described *Haliplus harminae* Vondel, 1990 from Hubei. Subsequently Vondel (1991, 1992, 1995, 1998, 2003a) described five new species from China and revised all known Chinese species. Makhan (1999) described further three new species from China, *Peltodytes aschnae* Makhan, 1999, *Haliplus rishwani* Makhan, 1999 and *Haliplus amrishi* Makhan, 1999, which were all treated as synonyms of *Peltodytes sinensis* (Hope, 1845), *Haliplus japonicus* Sharp, 1873 and *Haliplus sharpi* Wehncke, 1880 respectively by Vondel (2003b). Šťastný and Boukal (2003) described *Haliplus rejseki* Šťastný & Boukal, 2003 from Sichuan, being the first representative of the subgenus *Haliplidius* in China. Jia (2003) reported *Haliplus (Liaphlus) dalmatinus* Müller, 1900 (which identification is changed to *Haliplus abbreviatus* Wehncke, 1880 in this paper) and Jia (2003) and Vondel (2003b) reported *Peltodytes (Peltodytes) caesus* (Duftschmid, 1805) from China.

The studies of the material of the Haliplidae deposited in the Coleoptera collection of the Sun Yat-sen University in Guangzhou, China and in several European collections result in a considerable extension on the knowledge of Chinese Haliplidae. This justifies a review of the species known so far from China, specified to the provinces in which they are found. In the present paper one new species of *Haliplus* is described and other three species of *Haliplus* are reported as new for China. Including this study 29 species are now known from China.

## Material and methods

The present paper is based predominantly on 600 specimens of the family Haliplidae deposited in the Coleoptera collection of the Sun Yat-sen University (SYSU) in Guangzhou (Guangdong, China). All these specimens were identified by the first author and 25 specimens were re-examined by the second author. The second author examined another 210 specimens from the National Museum Prague, Czech Republic (NMPC); Naturhistorisches Museum Basel, Switzerland (NHMB), the Snow Entomological Collections of the University of Kansas, Lawrence, Kansas, USA (SEMC), the collection of F. Angelini, Brindisi, Italy (CA), the collection of H. Fery, Berlin, Germany (CF), the collection of A. Nilsson, Umeå, Sweden (CN) and second author's own collection (CV).

Details on distribution are partly adopted from Vondel (2005, 2007). The genera *Haliplus* and *Peltodytes* were redescribed in detail by Holmen (1987). The key to genera was given by Vondel (1995). Morphological terminology largely follows Holmen (1987), Vondel (1991, 1992, 1993) and Vondel et al. (2006). Photographs were taken using a Zeiss Axioskop 40 compound microscope and a Leica M205C stereomicroscope combined with AutoMontage software.

## Systematics

### Haliplus (Haliplus) latreillei sp. n.

urn:lsid:zoobank.org:act:1C3B87DA-E87E-40CA-B74D-2E537338AFEB

http://species-id.net/wiki/Haliplus_(Haliplus)_latreillei

Figs 1–6 

#### Type material.

 Holotype ♂, China, Guizhou, Guiyang, 6.x.1940, lgt. Zhe-long Pu (translation, labeled in Chinese) (SYSU). Paratypes (2 exs.): 1 ♂, same data as holotype (SYSU); 1 ♂, same data as holotype (NMW).

#### Description.

Length 2.9–3.0 mm, width 1.6–1.7 mm. Body oval, tapering backwards, widest before the middle (Fig. 1).

Head. Dark brown, somewhat lighter between eyes, anterior margin of clypeus densely punctured, but with much stronger and sparser punctures between eyes. Labrum yellowish brown with dark spot in the middle. Distance between eyes 1.6× width of one eye. Antennae light yellowish brown, not darkened apically. Palpi yellowish brown.

Pronotum. Yellow to yellowish brown. Without basal plicae, strongly and densely punctured. Lateral sides margined, straight to slightly convex. Base a little narrower than elytra at base.

Elytra. Yellowish brown, with dark interrupted lines on primary punctures rows, darkened along suture, with vague dark marks connecting primary puncture rows, without dark band basally. Completely margined. Primary puncture rows moderately strong and dense, 38–39 punctures in row 1. Secondary punctures moderately strong and dense along suture, moderately strong and much sparse on intervals. All punctures darkened.

Ventral side. Brown red, with legs and anterior 1/fifth of prosternal process yellow brown; elytral epipleura yellowish brown with strong darkened punctures, reaching to abdominal sternite 6. Prosternal process narrowed between coxae, grooved along each side, anterior edge not margined, with moderately strong punctation. Metaventral process slightly bulbous with a row of strong punctures on each side that is slightly impressed, else moderately punctured (Fig. 2). Metacoxal plates reaching to fifth sternite, moderately strongly punctured, near suture weakly punctured, row of setae on posterior edge (Fig. 3). Fifth and sixth abdominal sternite each with sparse transverse puncture row. Last abdominal sternite weakly punctured in apical portion. No setiferous striole present on dorsal face of hind tibia, longer tibial spur of hind legs with dense teeth on inner side, about 2/3× length of first metatarsomere.

Males. Pro- and mesotarsomeres moderately widened and provided with suction-pads. Mesotarsomere 1 not very strongly excised. Penis and parameres as Figs 4, 5, 6.

Female. Unknown.

**Figuress 1–6.**
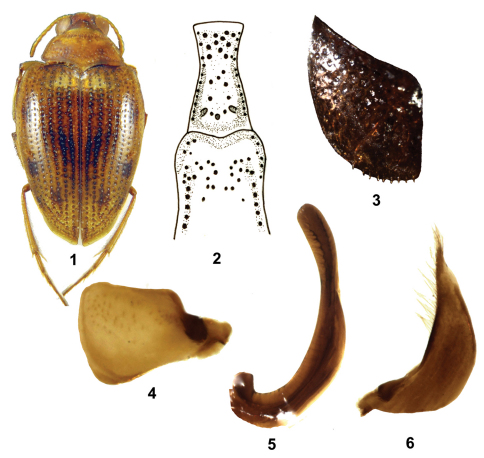
*Haliplus latreillei* sp. n. **1** habitus **2** prosternal and metaventral process **3** metacoxal plate **4** left paramere **5** penis **6** right paramere.

#### Etymology.

 The species is named in honour of Pierre André Latreille (1762–1833), a French entomologist who firstly used *Haliplus* as the genus name in 1802.

#### Differential diagnosis.

This species is close to *Haliplus japonicus* Sharp, 1873 and *Haliplus regimbarti* Zaitzev, 1908 in body size and shape, arrangement of elytral dark spots and black lines, punctuation and the row of setae on posterior edge of the metacoxal plates. However, the new species lacks pronotal basal plicae, its pronotum lacks the transverse basal rim and it differs from the above species in the shape of the median lobe and parameres of the aedeagus. Despite the absence of pronotal plicae this species clearly belongs to the subgenus *Haliplus* s. str. due to the absence of the metatibial setiferous striole.

#### Distribution.

 Only known from the type locality.

### List of Chinese Haliplidae

Only valid names are given in this list. For complete synonymy and distribution outside China see Vondel (2005)

#### 1. Haliplus (Haliplidius) confinis

Stephens, 1828

http://species-id.net/wiki/Haliplus_(Haliplidius)_confinis

Figs 7–10 

##### Material examined.

 **Xinjiang:** 1♂, Kashi, 1.viii.2006. lgt. Ling Zhao.

**Figuress 7–10.**
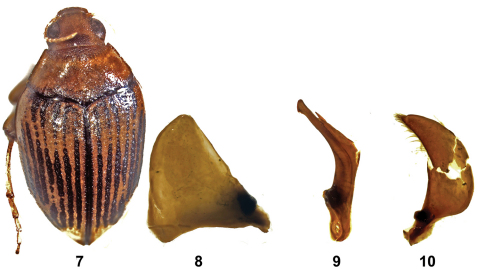
*Haliplus confinis* **7** habitus **8** left paramere **9** penis **10** right paramere.

##### Distribution.

 Widely distributed western and north-eastern Palaearctic species reaching the utmost north-west of China: Xinjiang. New to China.

#### 2. Haliplus (Haliplidus) rejseki

Šťastný et Boukal, 2003

http://species-id.net/wiki/Haliplus_(Haliplidus)_rejseki

##### Distribution.

 Endemic to China: Sichuan.

#### 3. Haliplus (Haliplus) aliae

Vondel, 2003

http://species-id.net/wiki/Haliplus_(Haliplus)_aliae

##### Distribution.

 Endemic to China: Tianjin.

#### 4. Haliplus (Haliplus) furcatus

Seidlitz, 1887

http://species-id.net/wiki/Haliplus_(Haliplus)_furcatus

##### Material examined.

 **Heilongjiang:** 2 exs., , Mishan, 26.viii.1964, lgt. De-ai Deng & Shou-fa Hou. **Inner Mongolia:** 12 exs., Hailar, 23–26.vii.2003, lgt. Feng-long Jia (2 exs. CV); 1 ex., Huhenuor, 21–25.vii.2005, lgt. Feng-long Jia.

##### Distribution.

 Western and north-eastern Palaearctic species reaching China in the north-east: Heilongjiang, Inner Mongolia. New record for Inner Mongolia.

#### 5. Haliplus (Haliplus) harminae

Vondel, 1990

http://species-id.net/wiki/Haliplus_(Haliplus)_harminae

##### Material examined.

 **Hunan:** 1 ♀., Nanyue Mt., 2.ix.1941, lgt. Zhe-long Pu; **Shaanxi:** 1 ♀., Chang’an, Weiqu, 12.viii.1984, lgt. Zhi-he Huang.

##### Distribution.

 Endemic to China: Hubei, Hunan, Shaanxi. New records for Hunan and Shaanxi.

#### 6. Haliplus (Haliplus) japonicus

Sharp, 1873

http://species-id.net/wiki/Haliplus_(Haliplus)_japonicus

##### Material examined.

 **Guizhou:** 4 exs., , Guiyang, 15.viii.1982, lgt. Zhi-he Huang; 1 ex., Guiyang, 6.x.1940, lgt. Zhe-long Pu; 1 ex., Guiyang, Pingba Horse farm, 13.viii.1982, lgt. Zhi-he Huang; 8 exs., Guiyang, Huaxi, 12.viii.1982, lgt. Zhi-he Huang (1 ex. CV). **Sichuan**: 3 exs., Emei Mt., 31.viii.1982, lgt. Zhi-he Huang (1 ex. CV). **Zhejiang:** 1 ex., Tianmushan Mt., 27–28.vii.2007, lgt. Feng-long Jia.

Additional material examined by Vondel: **Sichuan:** 1♂, Wenjian Distr., Guanxian Co., 56 km NW Changdu, Qingcheng Shan, 975 m, 30°53,8N, 103°32,8E, 13.vii.1999.lgt. A. Putz (CF). **Yunnan:** 15 exs., E. Weishan Mt., 1800–2500 m, 25°10'N, 100°21'E, 22–25.vi.1992, lgt. Vitkubáň (CV, NHMB); 36 exs. Shizong, 9–15.ix.2000, lgt. J. Bergsten (CN, CV).

##### Distribution.

 Eastern Palaearctic species, known from several provinces in the east and south of China: Beijing, Chongqing, Guizhou, Hunan, Jiangsu, Shanghai, Sichuan, Yunnan, Zhejiang. New records for Sichuan and Zhejiang.

#### 7. Haliplus (Haliplus) latreillei sp. n.

urn:lsid:zoobank.org:act:1C3B87DA-E87E-40CA-B74D-2E537338AFEB

http://species-id.net/wiki/Haliplus_(Haliplus)_latreillei

Figs 1–6 

##### Material examined.

See type material in Systematics chapter.

##### Distribution.

Endemic to China: Guizhou.

#### 8. Haliplus (Haliplus) regimbarti

Zaitzev, 1908

http://species-id.net/wiki/Haliplus_(Haliplus)_regimbarti

##### Material examined.

 **Fujian:** 1 ex., Nanjing County, Hexi town (in pool), 13.vii.2010, lgt. Feng-long Jia; 2 exs., Fuzhou, 14.vii.1958, lgt. Zhe-long Pu; 1 ex., same locality, 4.ix.1941. **Guangdong:** 15exs, , Guangzhou, Luogang, 20.iv.1958 (3 exs. CV); 11 exs., Danxiashan Mt., 27.v.2007, lgt. Feng-long Jia. **Guangxi:** 1 ex., Wuming, 17.vi.1977, lgt. Zhihe Huang; 2 exs., Yangshuo, 1985, lgt. Shou-jian Chen. **Henan:** 6 exs., , Xinyang, Jigongshan Mt., viii.1936; **Hubei:** 1 ex., , Wuchang, 17.v.1961, lgt. Zhe-long Pu. **Hunan:** 4 exs., Nanyue Mt., 4.ix.1941, lgt. Zhe-long Pu; 1 ex., Liyuan, 6.iii.1941, lgt. Zhe-long Pu; 2 exs., Shaanxi, Chang’an, Weiqu, 21.viii.1984, Zhi-he Huang. **Jiangxi:** 1 ex., , Jinggangshan, Ciping, 18.ix.2010, lgt. Shuang Zhao; 1 ex., Jiangxi, Jinggangshan, Jingzhushan Mt., 4.x.2010, lgt. Feng-long Jia; 24 exs., same locality, 25.iv.2011, lgt. Fenglong Jia & Shuang Zhao; 11 exs. Lushan, 10.viii.1963, lgt. Zhe-long Pu; 2 exs., Lushan, 10.viii.1963, lgt. Zhe-long Pu. **Yunnan:** 3 exs., , Pohui, 9.x.1940, lgt. Zhe-long Pu.

Additional material examined by Vondel: **Guizhou:** 1 ex., Fodingshan, ganshi, 25 km S Shiquian, 1300 m, 5–9.vi.1997, lgt. Bolm; 4 exs. Leigongshan, Xijiang, 1200–1900 m, 29.v-2.vi.1997, lgt. Bolm (CV, NHMB). **Jiangxi:** 1♀, “Sharp; China, Kia Kiang; Dr. Régimbart vidit 1898; **Shaanxi**: 1 ex., without precise locality data; 1♀, Süd Schensi [no further locality data] (NMPC).

##### Distribution.

 Endemic and widespread in the south-eastern part of China: Anhui, Fujian, Guangdong, Guangxi, Guizhou, Henan, Hubei, Hunan, Jiangsu, Jiangxi, Shandong, Shaanxi, Taiwan, Yunnan, Zhejiang. New records for Guangxi, Hubei, Shaanxi and Yunnan.

#### 9. Haliplus (Haliplus) ruficollis

(De Geer, 1774)

http://species-id.net/wiki/Haliplus_(Haliplus)_ruficollis

Figs 11–14 

##### Material examined.

 **Xinjiang**: 2♂♂, Yining, Yili river valley, 28.vii.2005, lgt. Ling Zhao (1 ex., CV); 2♂♂, Kanasi lake, 8.viii.2005, lgt. Ling Zhao; 1♂, 2♀♀, Tacheng, Hardun river bank, 4.viii.2005, lgt. Ling Zhao.

##### Distribution.

 A widespread western Palaearctic species reaching China in the utmost north-western part: Xinjiang. New to China.

**Figure:**
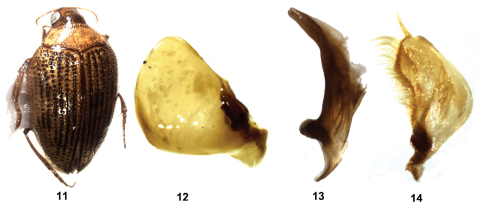
**Figuress 11–14.** *Haliplus ruficollis* **11** habitus **12** left paramere **13** penis **14** right paramere.

#### 10. Haliplus (Haliplus) sibiricus

Motschulsky, 1860

http://species-id.net/wiki/Haliplus_(Haliplus)_sibiricus

Figs 15–18 

##### Material examined.

 **Xinjiang:** 32 exs., Yili Agricultral School, 3.viii.1964, lgt. Zhihe Huang (3 exs., CV); 9 exs., suburb of Wulumuqi, 16.vii.1984, lgt. Zhihe Huang; 6 exs., Wulumuqi, Liudaowan, 3.vii.1984, lgt. Zhihe Huang; 19 exs., Hongxing Farm, 6.viii.2005; 19 exs., Hot Spring, Swamp, 24.vii.2005, lgt. Ling Zhao; 3 exs., Gongnaisigou, 30.vii.2005; 7 exs., Altai, Xiaodonggou, 10.viii.2005, lgt. Ling Zhao; 5 exs. Tacheng, Hardun river bank, 4.viii.2005, lgt. Ling Zhao; 5 exs., Huocheng, river weir and river bank, 4.viii.2005, lgt. Ling Zhao; 4 exs., Kanasi lake, 8.viii.2005, lgt. Ling Zhao (2 exs., CV); 2 exs., Nalati steppe, 30.vii.2005, lgt. Ling Zhao.

Additional material examined by Vondel: **Qinghhai:** 6 exs. prov. Huangzhong env. Taer [lamasery], 36°28.8-29.5'N, 101°34.0-34.1'E, 2665–2780 m, 17.vii.2005, lgt. J. Hájek, D. Král & J. Růžička leg. (CV, NMPC)

**Figure:**
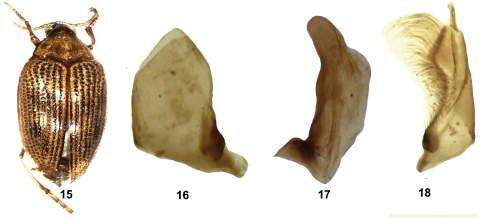
**Figuress 15–18.** *Haliplus sibiricus* **15** habitus **16** left paramere **17** penis **18** right paramere.

##### Distribution.

 A widely spread western and north-eastern Palaearctic species, reaching the west of China: Qinghai, Xinjiang. New to China.

#### 11. Haliplus (Haliplus) simplex

Clark, 1863

http://species-id.net/wiki/Haliplus_(Haliplus)_simplex

##### Material examined.

 **Heilongjiang:** 1 ex., Liangshui, last third of July, 1977; 3 exs., Harbin, swampland, 29.vii.1962, lgt. Zhe-long Pu; 22 exs., same locality, 23.vii.1962; 1 ex., Harbin, 26.viii.1962, lgt. Zhe-long Pu; 3 exs., Wudalianchi, 6–10.viii.2008, lgt. Feng-long Jia. **Inner Mongolia:** 1 ex., Hailar, 23–26.vii.2003. **Jilin:** 2 exs., Gongzhuling, 5.viii.1962, lgt. Zhe-long Pu. **Shaanxi:** 16 exs., Chang’an, Weiqu, 21.viii.1984, lgt. Zhi-he Huang.

Additional material examined by Vondel: **Anhui:** 1 ex., [no further locality data], lgt. P. Brinck (CN). **Inner Mongolia:** 3 exs., North Manchuria, Djalantun St. [Buth Qi], 6.v.1939, lgt. P. Brinck (CN).

##### Distribution.

 Eastern Palaearctic species, known from several provinces in the eastern part of China: Anhui, Beijing, Guangdong, Heilongjiang, Inner Mongolia, Jiangsu, Jilin, Liaoning, Shaanxi, Shandong, Zhejiang. New records for Anhui, Inner Mongolia and Jilin.

#### 12. Haliplus (Haliplus) steppensis

Guignot, 1954

http://species-id.net/wiki/Haliplus_(Haliplus)_steppensis

##### Material examined.

**Heilongjiang:** 1 ex, Liangshui, last third of July 1977. **Inner Mongolia:** 7 exs., Jining suburb, autumn, 1975 (2 exs., CV); 1 ex., Linhe, autumn, 1975; 3 exs., Hailar, 23–26.vii.2003, lgt. Feng-long Jia.

##### Additional material examined by Vondel:

**Gansu:** 15 exs., Dagcanglhamo [=Langmusi] env., 34°04.7'N, 102°38.1'E, 3465 m alt, 23–25.vi.2005, leg. J. Hájek, D. Král & J. Růžička (CV, NMPC). **Qinghai:** 4 exs., Haibu env. 3190–3270 m. 36°48.4–49.8'N, 100°45.4–49.7'E, 13–15.vii.2005, leg. J. Hájek, D. Král & J. Růžička; 8exs., 7 km NE Ulan, 3020 m alt. 36°57.6'N, 98°30.6'E, 7.vii.2005, leg. J. Hájek, D. Král & J. Růžička (CV, NMPC).

##### Distribution.

 North-eastern Palaearctic species, known from the north of China: Gansu, Heilongjiang, Inner Mongolia, Qinghai. New records for Gansu, Inner Mongolia and Qinghai.

#### 13. Haliplus (Liaphlus) abbreviatus

Wehncke, 1880

http://species-id.net/wiki/Haliplus_(Liaphlus)_abbreviatus

##### Material examined.

 **Xinjiang** 1♂, , Kuerle, Shayidong, 22.vii.1984, lgt. Zhihe Huang ; 1♂, Kashi, 1.VIII.2006, lgt. Ling Zhao ; 1♀, Hetian, 16.vii.2006, lgt. Ling Zhao; 4♂♂, 5♀♀, Shule, 12.viii.2006, lgt. Ling Zhao (1♂, 1♀, CV).

##### Note.

The specimen from Kuerle was identified as *Haliplus dalmatinus* Müller, 1900 and reported as new to China by Jia (2003). The reexamination of this specimen shows that its previous identification was incorrect. *Haliplus dalmatinus* is removed from the Chinese list

##### Distribution.

 Southern Palaearctic reaching the western part of China: Xinjiang.

#### 14. Haliplus (Liaphlus) basinotatus

Zimmermann, 1924

http://species-id.net/wiki/Haliplus_(Liaphlus)_basinotatus

##### Material examined.

 **Heilongjiang:** 3 exs., Mishan, 26.viii.1964, lgt. De-ai Deng & Shou-fa Hou; 1 ex., Harbin, 28.vii.1962, lgt. Zhe-long Pu.

Additional material examined by Vondel: 1 ex. Djalantun St., 6.v.1939. lgt. P. Brinck [Butha Qi, Inner Mongolia] (CN)

##### Distribution.

North-eastern Palaearctic species, reaching China in the north-east: Heilongjiang, Inner Mongolia, Jilin, Liaoning.

#### 15. Haliplus (Liaphlus) chinensis

Falkenström, 1932

http://species-id.net/wiki/Haliplus_(Liaphlus)_chinensis

##### Material examined.

 **Guizhou**: 1 ex., Pingba Horse Farm, 13.vii.1982, lgt. Zhi-he Huang. **Sichuan:** 4 exs., , Dakang, 14.viii.2001, lgt. Ling Zhao.

Additional material examined by Vondel: **Xinjiang:** 1♀, Chingkiang, xi.1877 (NMPC). **Yunnan:** 5 exs., Shizong, 11–15.ix.2000, lgt. J. Bergsten (CN, CV)

##### Note.

The older records of *Haliplus ovalis* Sharp, 1884 from China likely concern *Haliplus chinensis* (for details see Vondel 1991, 2005)

##### Distribution.

 Endemic to China, known from several provinces from west to east: Beijing, Fujian, Guizhou, Inner Mongolia, Jiangsu, Shandong, Shanghai, Sichuan, Shanxi, Xinjiang, Yunnan, Zhejiang.

#### 16. Haliplus (Liaphlus) davidi

Vondel, 1991

http://species-id.net/wiki/Haliplus_(Liaphlus)_davidi

##### Material examined.

 **Tianjin:** 1 ex., Nanda, iv.1956.

##### Distribution.

 Eastern Palaearctic and Oriental species, known from the north and south of China: Beijing, Heilongjiang, Tianjin, Yunnan. New record for Tianjin.

#### 17. Haliplus (Liaphlus) diruptus

J. Balfour-Browne, 1947

http://species-id.net/wiki/Haliplus_(Liaphlus)_diruptus

##### Material examined.

 **Guizhou:** 1 ex., Guiyang, 9.x.1940, lgt. Zhe-long Pu. **Heilongjiang:** 1 ex., Wudalianchi, 6–10.viii.2008, lgt. Feng-long Jia. **Hunan:** 3 exs., Yizhang, 1.iv.1942, lgt. Zhe-long Pu; 2 exs., same locality, 10.ii.1941.**Shaanxi:** 1 ex., Chang’an, Wutaishan Mt., 23.viii.1984.

Additional material examined by Vondel: **Anhui:** 1 ex. Anhui [without further locality data], lgt. P. Brinck (CN). **Guizhou:** 1 ex., Fodingshan, Ganshi, 25 km S. Shiquian, 1300 m, 5–9.vi.1997, lgt. Bolm (NHMB). **Yunnan:** 1♂, 25 km E Zhongdian, 3300–4000 m, 12–14.vii.1995, lgt. Bolm (NHMB); 9 exs. Shizong, 10–13.ix.2000, lgt. J. Bergsten (CN, CV).

##### Distribution.

 Eastern Palaearctic and Oriental species, widely spread in the eastern part of China: Anhui, Beijing, Fujian, Guizhou, Hainan, Heilongjiang, Hong Kong, Hubei, Hunan, Jiangsu, Liaoning, Shaanxi, Shandong, Shanghai, Taiwan, Tianjin, Yunnan. New records for Anhui and Shaanxi.

#### 18. Haliplus (Liaphlus) excoffieri

Vondel, 1991

http://species-id.net/wiki/Haliplus_(Liaphlus)_excoffieri

##### Material examined.

 **Guizhou:** 1 ex., , Pingba Horse Farm, 13.vii.1982, lgt. Zhi-he Huang. **Yunnan:** 1 ex., Pohui, 9.x.1940, lgt. Zhe-long Pu.

Additional material examined by Vondel: **Yunnan:** 5 exs. Shizong, 12–13.ix.2000, lgt. J. Bergsten (CN, CV).

##### Distribution.

 Endemic species in the south of China: Guizhou, Yunnan. New record for Guizhou.

#### 19. Haliplus (Liaphlus) eximius

Clark, 1863

http://species-id.net/wiki/Haliplus_(Liaphlus)_eximius

##### Material examined.

 **Guangdong:** 2 exs., Xingning, Luofu, Tieshan, 1.vii.2004, lgt. Feng-long Jia. **Guizhou:** 3 exs., Guiyang, Huaxi, 12.viii.1982, lgt. Zhi-he Huang. **Xinjiang:** 1 ex., Shule, 12.viii.2006, lgt. Ling Zhao. **Yunnan:** 1 ex., Lufeng village, 26.iii.1940, lgt. Zhe-long Pu.

Additional material examined by Vondel: **Xinjiang:** 1 ♂, Chingkiang, xi.1877 [no further data](NMPC).

##### Distribution.

 Eastern Palaearctic and Oriental species, known from the west, east and south-east of China: Beijing, Fujian, Guangdong, Guizhou, Hunan, Jiangsu, Liaoning, , Shanghai, Sichuan, Xinjiang, Yunnan, Zhejiang. New record for Yunnan.

#### 20. Haliplus (Liaphlus) holmeni

Vondel, 1991

http://species-id.net/wiki/Haliplus_(Liaphlus)_holmeni

##### Distribution.

 Endemic to China: Yunnan.

#### 21. Haliplus (Liaphlus) kotoshonis

Kano & Kamiya, 1931

http://species-id.net/wiki/Haliplus_(Liaphlus)_kotoshonis

##### Distribution.

 Eastern Palaearctic/Oriental species, known from the Ryukyu Islands (Japan) and Taiwan.

#### 22. Haliplus (Liaphlus) pulchellus

Clark, 1863

http://species-id.net/wiki/Haliplus_(Liaphlus)_pulchellus

##### Material examined.

 **Guangxi**: 2 exs., Nanning, 22.vi.1958, lgt. Zhe-long Pu.

##### Distribution.

 Oriental species, reaching China in the south: Fujian, Guangxi. New record for Guangxi.

#### 23. Haliplus (Liaphlus) sharpi

Wehncke, 1880

http://species-id.net/wiki/Haliplus_(Liaphlus)_sharpi

##### Material examined.

(examined by Vondel): **Anhui:** 3 exs., Anhui, lgt. P. Brinck [no futher data] (CN). **Guizhou:** 1♂, Fodingshan, Ganshi, 25 km A Shiquian, 1300 m, 5–9.vi.1997, lgt. Bolm (NHMB). **Yunnan:** 6 exs., Shizong, 10–13.ix.2000, lgt. J. Bergsten (CN, CV)

##### Distribution.

 Eastern Palaearctic species, known from several provinces in the east and south of China: Anhui, Chongqing, Fujian, Guizhou, Liaoning, Shanghai, Taiwan, Yunnan.

#### 24. Peltodytes (Peltodytes) caesus

(Duftschmid, 1805)

http://species-id.net/wiki/Peltodytes_(Peltodytes)_caesus

##### Material examined.

 **Xinjiang:** 1 ex., Yili Agriculture School, 3.viii.1984, lgt. Zhi-he Huang; 1 ex., Yining, Yili river valley, 28.vii.2005; 1ex., Emin, Hongxing Farmland, 6.viii.2005, lgt. Ling Zhao (1 ex., CV); 1 ex., Tacheng, Hardun river bank, 4.viii.2005, lgt. Ling Zhao.

##### Distribution.

 Widely spread Palaearctic species, reaching to China in the utmost north-west: Xinjiang.

#### 25. Peltodytes (Peltodytes) coomani

Peschet, 1923

http://species-id.net/wiki/Peltodytes_(Peltodytes)_coomani

##### Material examined.

**Guangdong:** 1 ex., Guangzhou, Kangle (Sun Yat-sen University campus), iv.1959, lgt. Ping Lin; 1 ex., same locality, 24.vii.1964, lgt. Jiu-ru Zhang. **Guangxi**: 1 ex., Nanning, iv.1959, lgt. Zhe-long Pu; 2 ex., Nanning, vi.1958, lgt. Zhe-long Pu (2 ex., CV). **Hainan:** 1 ex., Dongfang, Huangliu, 25.xii.1963, lgt. Tong-xu Peng; 2 exs., Tongshi, 27.xii.1963, lgt. Tong-xu Peng; 1 ex., Wanning County, Xinglong, 3.i.1964, lgt. Tong-xu Peng; 2 exs., Jianfengling Mt., 19.xii.1963, lgt. Tong-xu Peng.

##### Distribution.

Oriental species, reaching to China in the south-east: Guangdong, Guangxi, Hainan. New record for Guangxi.

#### 26. Peltodytes (Peltodytes) dauricus

Zimmermann, 1924

http://species-id.net/wiki/Peltodytes_(Peltodytes)_dauricus

##### Material examined.

 **Heilongjiang**: 1♂, Mishan, 1.x.1959.

Additional material examined by Vondel: **Inner Mongolia:** 5 exs., Djalantun [Buth Qi], 6.v.1939, lgt. P. Brinck (CN). **Heilongjiang:** 3 exs., Harbin, Manchoukuo, 4.x.1937, lgt. M.A. Weymarn; 1 ex., Harbin, Manchuria, 1938, lgt. M.I. Nikitin (SEMC). **Liaoning:** 1 ex., Korea, Daireu [=Dalian Shi, Liaoning], 1–15.ix.1937, lgt. Weymarn (CN).

##### Distribution.

 North-eastern Palaearctic species, reaching to China in the north-east: Heilongjiang, Inner Mongolia, Liaoning.

#### 27. Peltodytes (Peltodytes) intermedius

(Sharp, 1873)

http://species-id.net/wiki/Peltodytes_(Peltodytes)_intermedius

##### Material examined.

 **Sichuan:** 1 ♂, Emeishan Mt., 6.viii.1982, lgt. Zhi-he Huang. **Guangdong:** 1♂, Guangzhou, Luogang cave, 12.x.1932, lgt. Y.W. Diou.

##### Distribution.

 Eastern Palaearctic species, known from the south-east of China: Beijing, Fujian, Guangdong, Shanghai, Sichuan, Taiwan, Zhejiang. New records for Sichuan and Guangdong.

#### 28. Peltodytes (Peltodytes) pekinensis

Vondel, 1992

http://species-id.net/wiki/Peltodytes_(Peltodytes)_pekinensis

##### Material examined.

 **Guangdong:** 1 ex., the second worker’s culture palace, x.1955. **Shaanxi**: 4 exs. Xi’an Chanba river, 11.v.2011, lgt. Fenglong Jia; 6 exs. same locality, lgt. Hájek (NMPC). **Tianjin:** 1 ex., Nanda, iii.1956.

##### Distribution.

 Eastern Palaearctic species, known from the most-eastern part of Russia and several provinces in the east of China: Beijing, Fujian, Guangdong, Hebei, Liaoning, Shaanxi, Shandong, Tianjin. New records for Guangdong, Shaanxi and Tianjin.

#### 29. Peltodytes (Peltodytes) sinensis

(Hope, 1845)

http://species-id.net/wiki/Peltodytes_(Peltodytes)_sinensis

##### Material examined.

 **Chongqing:** 1 ex., Nanchuan, Tianxing, 27.vii.2003, lgt. Jian-hua Huang. **Fujian:** 1 ex., , Fuzhou, 14.vii.1956, lgt. Zhe-long Pu; 1 ex., Fuzhou, Gushan, 14.vii.1956, lgt. Li-zhong Hua; 7 exs., Fuzhou, Xihu, 3.xi.1963, lgt. Shan-xiang Lin. **Guangdong:** 1 ex., Lianxian, vi.1945, lgt. Zhe-long Pu; 1 ex., Shaoguan, Yingde, 4.viii.1962, lgt. Ping Lin; 1 ex., Sihui, Dasha, 6.iii.1998, lgt. Feng-long Jia; 1 ex., Heshan, 18.3.1994, lgt. Feng-long Jia; 1 ex., Lianshan County, Shangshuai, 4.v.2000, lgt. Feng-long Jia; 1 ex., Honan Island, 19.iii.1938, lgt. Chiu-an Wang; 1 ex., Zhaoqing, 12.x.1974, Meiying Wang; 1 ex., Qujiang, date for collection could not be read, lgt. Zhe-long Pu; 1 ex., Guangzhou, Ershatou, 11.iv.1958; 2 exs., Fengkai, Heishiding Mt., 29.v.1984, lgt. Zhi-he Huang; 3 exs., Lianxian, Dadongshan Mt., 16.ix.1993, lgt. Feng-long Jia; 11 exs., Xingning, Luofu, Tieshan, 1.vii.2004, lgt. Feng-long Jia; 1 ex., Danxiashan Mt., 23.v.2008, lgt. Feng-long Jia; 1 ex., Puning, 1958, lgt. Zhe-long Pu; 15 exs., Huaxian (Huadu) Dabuling, 26.viii.1983, lgt. Zhi-he Huang; 2 exs., Guangzhou, Baiyunshan, 18.iv.1958, lgt. Zhe-long Pu. **Guangxi:** 6 exs., , Yangshuo, 1958, lgt. Shoujian Chen et. al.; 1 ex., Longlin, 22.v.1977, lgt. Zhi-he Huang; 4 exs., Wuming, 7.vi.1977, lgt. Zhi-he Huang; 1 ex.m Shangsi, 24.vii.1977, lgt. Zhi-he Huang; 1 ex., Huaping, 25.vi.1974, lgt. De-xiang Gu; 1 ex., Jingxi, Bangliang, 31.vii.2010, lgt. Jian-hua Huang. **Guizhou:** 1 ex., Guiyang, 6.x.1940, lgt. Zhe-long Pu; 3exs., Guiyang, 12.viii.1982, lgt. Zhi-he Huang; 3 exs., Pingba Horse Farm, 13.viii.1982, lgt. Zhi-he Huang (1 ex., CV); 1 ex., Guiyang, 15.viii.1982, lgt. Zhi-he Huang; 1 ex., Fanjingshan, half of mount, 29.vii.2001, lgt. Hong Pang; 1 ex., Rongxian County, Pingyang, Xiaodanjiang river, 685m, 15.ix.2005, lgt. Shuang Zhao; 2 exs., , Guiyang, 6.x.1940, lgt. Zhe-long Pu. **Hubei:** 18 exs., Zigui, Jiutouling, 150m, 20.vii.1993, lgt. Xing-ke Yang & Wen-zhu Li; 2 exs., Badong, Sanxia Forest workshop, 160m, 30.vii.1993, lgt. Xing-ke Yang & Wen-zhu Li; 1 ex., Wuchang, vi.1958, lgt. Jieyue Hu. **Hunan:** 2 exs., Nanyue Mt., 4.ix.1941, lgt. Zhe-long Pu; 16 exs., Daotong, 19.viii.1982, lgt. Zhi-he Huang; 1ex., Huaihua, 17.viii.1982, lgt. Zhi-he Huang; 1 ex., Yizhang, 8.x.1941, lgt. Zhe-long Pu; 1 ex., same locality, 10.ii.1941; 10 exs., Huaihua, Yushuwan, 17.vi.1965, lgt. Zhen-yao Chen; 4 exs., Qingjiang, Anjiang, 20.vi.1956, lgt. Zhen-yao Chen. **Jiangxi:** 2 exs., Jingu County, 22.viii.1974; 6 exs., Nanchang, viii.1957, lgt. Xi-wen Chen; 2 exs., samelocality, 26.viii.1963, lgt. Zhe-long Pu; 2 exs., Shangrao, Sanqingshan Mt., 15–20.iv.2007, lgt. Feng-long Jia; 2 exs., Longnan, Jiulianshan Mt., 12–13.vii.2008, lgt. Feng-long Jia; 3 exs., Jiujiang, Changdu, Linshan, 15–20.viii.2010, lgt. Yan Mei; 1 ex., Lushan, Poyang Lake, 10.viii.1963, lgt. Zhe-long Pu; 1ex., Nanchang, no other data; 9 exs., Yongxin County, 19.viii.1974; 3 exs., Fuzhou, 16–18.viii.1974; 1 ex., , Ji’an, 13.viii.1974; 1 ex., Jinggangshan, 16–18.viii.1974; 2 exs., Jinggangshan, Shuangxikou, 13.x.2010, lgt. Feng-long Jia; 4 exs. Jinggangshan, Baiyinhu, 800 m, 27.iv.2011. **Shaanxi:** 17 exs., Chang’an Weiqu, 21.viii.1984, lgt. Zhi-he Huang; 1ex., Xi’an Wujiafen, 17.viii.1984, lgt. Zhi-he Huang; 2 exs., South of Chang’an, Wutai, 23.viii.1984, lgt. Zhi-he Huang; 1 ex., Zhenba, 20.vii.1975, lgt. Shuzhi Ren. **Sichuan:** 3 exs., Qingchengshan Mt., 8.viii.1982, lgt. Zhi-he Huang; 7 exs.,Wanxian, Longju, 450m, 14.vii.1993, lgt. Wen-zhu Li; 4 exs., Wanxian, Wang’erbao, 1200m, 9.vii.1993, lgt. Wen-zhu Li; 1 ex., Fengdu, 200m, 1.vi.1994, You-wei Zhang; 8 exs., Emeishan Mt., 31.vii.1983, lgt. Zhi-he Huang ( 3 exs., CV); 2 exs., Dakang, 14.viii.2001, lgt. Ling Zhao; 1 ex. Xi’an, Chanba river, 11.iv.2011, lgt. Fenglong Jia; 3 exs. same data, but J. Hájek lgt. (NMPC). **Tianjin:** 1 ex., Nanda, iv.1956. **Yunnan:** 4exs., Pohui, 9.x.1940, lgt. Zhe-long Pu; 1 ex., Lijiang, v.2007, lgt. Run-lin Xu.

Additional material examined by Vondel: **Anhui:** 3 exs.,Anhui [no further data], lgt. P. Brinck (CN). **Fujian:** Kuatun, 1946 (NHMB). **Guangxi:** 1 ex., 20 km N Lingchuan, 500 m, 21–24.vi.1997, lgt. Bolm (NHMB). **Jiangxi:** 1 ex., 5 km N Daduan town, 114°35'53"E, 28°36'30"N, ca 450 m, 29.iii.2003, lgt. Schönmann, Komarek & Wang (NMW). **Shanghai:** 1 ex.,Kiangsee Jangtzi, Shanghai Xanthus [no further data] (CA). **Yunnan:** 73 exs. Shizong, 9–15.ix.2000, lgt. J. Bergsten (CN, CV)

##### Distribution.

 Eastern Palaearctic and Oriental species, widely spread in the eastern and south-eastern part of China: Anhui, Beijing, Chongqing, Fujian, Guangdong, Guangxi, Guizhou, Hainan, Hebei, Henan, Hubei, Hunan, Jilin, Jiangsu, Jiangxi, Liaoning, Shaanxi, Shandong, Shanghai, Sichuan, Taiwan, Yunnan, Zhejiang. New records for Chongqing, Hubei and Shaanxi.

## Supplementary Material

XML Treatment for Haliplus (Haliplus) latreillei

XML Treatment for Haliplus (Haliplidius) confinis

XML Treatment for Haliplus (Haliplidus) rejseki

XML Treatment for Haliplus (Haliplus) aliae

XML Treatment for Haliplus (Haliplus) furcatus

XML Treatment for Haliplus (Haliplus) harminae

XML Treatment for Haliplus (Haliplus) japonicus

XML Treatment for Haliplus (Haliplus) latreillei

XML Treatment for Haliplus (Haliplus) regimbarti

XML Treatment for Haliplus (Haliplus) ruficollis

XML Treatment for Haliplus (Haliplus) sibiricus

XML Treatment for Haliplus (Haliplus) simplex

XML Treatment for Haliplus (Haliplus) steppensis

XML Treatment for Haliplus (Liaphlus) abbreviatus

XML Treatment for Haliplus (Liaphlus) basinotatus

XML Treatment for Haliplus (Liaphlus) chinensis

XML Treatment for Haliplus (Liaphlus) davidi

XML Treatment for Haliplus (Liaphlus) diruptus

XML Treatment for Haliplus (Liaphlus) excoffieri

XML Treatment for Haliplus (Liaphlus) eximius

XML Treatment for Haliplus (Liaphlus) holmeni

XML Treatment for Haliplus (Liaphlus) kotoshonis

XML Treatment for Haliplus (Liaphlus) pulchellus

XML Treatment for Haliplus (Liaphlus) sharpi

XML Treatment for Peltodytes (Peltodytes) caesus

XML Treatment for Peltodytes (Peltodytes) coomani

XML Treatment for Peltodytes (Peltodytes) dauricus

XML Treatment for Peltodytes (Peltodytes) intermedius

XML Treatment for Peltodytes (Peltodytes) pekinensis

XML Treatment for Peltodytes (Peltodytes) sinensis
